# Preparation and Preliminary Evaluation of Dual-functional Nanoparticles for MRI and siRNA Delivery

**DOI:** 10.22037/ijpr.2021.115099.15219

**Published:** 2021

**Authors:** Jingxin Sun, Yuanfei Zhou, Guangyu Jin, Yong Jin, Jishan Quan

**Affiliations:** a *Department of Pharmaceutics, College of Pharmacy, Yanbian University, Yanji 133000, China. *; b *Department of Radiology, Yanbian University Hospital, Yanji 133000, China.*; 1 *JS and YZ contributed equally to this work.*

**Keywords:** Lipid polycation, Superparamagnetic iron oxide nanoparticles, Gene silencing, siRNA, Magnetic resonance imaging

## Abstract

In order to improve the transfection efficiency of gene vectors and monitor the effect of gene therapy, this study prepared a drug delivery vector with dual functions. The thiourea reaction was used to synthesize polyethyleneimine (PEI, MW: 1.8 kDa) with superparamagnetic iron oxide nanoparticles (SPION) (PEI1800-SPION), and the lipid polycationic gene vector PEI1800-SPION loaded cationic liposome (LP-PEI1800-SPION) was further prepared by ethanol injection method. Agarose gel electrophoresis experiment, cytotoxicity experiment, and *In-vitro* gene silencing experiment were used to evaluate the vector and screen the optimal prescription of LP-PEI1800-SPION/siRNA. When the weight ratio of LP-PEI1800-SPION to siRNA is 20, the transfection efficiency of the nanoparticles was the highest, and the silencing efficiency of the target protein was the largest. The cytotoxicity of LP-PEI1800-SPION was low; when the concentration was in the range of 1-50 μg/mL, the survival rate of the four types of cells was above 80%. Prussian blue staining experiments and *In-vitro* MRI imaging experiments showed that cells had significant uptake and imaging capabilities for LP-PEI1800-SPION. In conclusion, the visualized polycationic lipid siRNA delivery vehicle (LP-PEI1800-SPION) was successfully prepared in this experiment, which provides a research basis for further theranostics of liver cancer.

## Introduction

Cancer, also known as a malignant tumor, is one of the major diseases that endanger human health. Its fatality rate is second only to cardiovascular disease. Its treatment has always been a problem in the field of medical research (1). The traditional treatment of malignant tumors often has limited inhibitory effects on advanced and metastasized tumors, is not selective, and hence tends to cause great damage to normal tissues and organs and body functions (2). With the development of molecular biology and biotechnology and the completion of the Human Genome Project, the discovery of gene therapy has provided new perspectives for the treatment of a variety of diseases such as genetic diseases, malignant tumors, cardiovascular diseases, and autoimmune diseases (3). Gene silencing mediated by small interfering RNA (siRNA) is rapidly emerging. Due to the high efficiency and specificity of siRNA in inhibiting gene expression, it has received great attention from researchers. However, naked siRNA has a very short half-life in the blood circulation. To exert a silencing effect, multiple physiological obstacles need to be overcome, such as the degradation by serum nucleases, the clearance by macrophages in the blood, and the degradation by immune cells or extracellular matrix (4). Therefore, the development of a safe and efficient gene carrier to deliver siRNA into target cells is key to the success of effective gene silencing *in-vivo*.

Liposomes are microvesicles with a phospholipid bilayer structure. The cationic liposomes can be electrostatically combined with negatively charged siRNA molecules and then enter the target cell through membrane fusion and endocytosis, after which they release the siRNA, affecting target gene expression. Another gene delivery system—cationic polymers—has also been studied. It has the advantages of good siRNA binding and protection, good biocompatibility, and easy modification. According to the intracellular microenvironments, polymers with different functional components can be designed to meet different drug delivery requirements. In recent years, it has been proposed to combine liposomes with cationic polymers to form new nanoparticles for gene delivery to further improve their gene delivery (5, 6). Pinnapireddy (7) combined liposomes with low-molecular-weight polyethyleneimine (PEI) and found that the resulting carrier complex has low cytotoxicity, high transfection efficiency, and good storage stability.

Magnetic resonance imaging (MRI) is a non-invasive, non-radiative, and high-resolution imaging technique (8). Superparamagnetic iron oxide nanoparticles (SPION) are an ideal MRI Transverse relaxation (T2) negative contrast agent. SPION can reduce the signal intensity by shortening the T2 relaxation time, making the image show negative contrast, thereby increasing the difference between healthy tissue and pathological tissue and improving diagnostic accuracy. The combination of siRNA gene silencing technology and imaging technology can realize visualized gene delivery, release, and accumulation *in-vivo*. At the same time, through tissue imaging, we can study the disease process more accurately and adjust the treatment plan in time, effectively improving treatment efficiency and realizing individualized treatment and precision medicine. Shen (9) constructed an MRI visual delivery vector Polyethylene glycol-grafted polyethyleneimine (PEG-g-PEI) containing neuroblastoma-specific ligand GD2 ganglioside. The Bcl-2-siRNA (*Bcl-2 *gene is a key apoptosis inhibitor that is overexpressed in many tumors) carried by this vector can specifically promote apoptosis of Neuroblastoma (NB) tumor cells. MRI studies show that the magnetic signal intensity in the target cell is significantly reduced, which proves that the carrier has the potential to be used in the field of MRI visualization of siRNA silencing and anti-tumor activity. Wu (8) synthesized a new type of folic acid (FA)-functionalized SPION with cationic amylose (CA-SPION) (FA-CA-SPION) as the backbone. The experimental results showed that FA-CA-SPION could mediate cell-specific uptake of Survivin-siRNA, leading to a significant downregulation of survivin in liver cancer cells. *In-vitro*, MRI results showed that with increasing iron concentrations in nanoparticles, the T2 signal intensity gradually decreases, and the multifunctional carrier achieves targeted siRNA delivery and visualizes the drug delivery process in liver cancer cells.

Herein, we constructed a siRNA delivery carrier (LP-PEI1800-SPION) for MRI visualization in order to examine whether cationic liposomes (LP) and polymer (PEI1800) exert a synergistic effect in gene transfer. In addition, PEI1800 was used to modify SPION and increase its stability. The optimal prescription was investigated through *in-vitro* experiments, and the feasibility of the carrier in MRI diagnosis was preliminarily explored.

## Experimental

*Materials*

Polyethyleneimine(PEI, MW 1800) was purchased from Sigma-Aldrich (USA). 1,2 Dioleoyl-3-trimethylammoniumpropane (DOTAP), 1,2-Dioleoyl-sn-glycero-3-phosphoethanolamine (DOPE), and cholesterol were purchased from Avanti Polar Lipids, Inc (Alabaster, AL, USA). Tris, Prussian Blue Staining Kit, Agarose, ethidium bromide (EtBr), RNase free water (DEPC water), Kanamycin, Ampicillin，Ethylenediaminetetraacetic acid disodium salt dihydrate (Na2·EDTA·2H_2_O)，N’-a-hydroxythylpiperazine-N’-ethanesulfanic acid (HEPES) and Glucose were purchased from Solarbio (Beijing, China). Cell Culture Lysis Reagent, Luciferase Reporter 1000 Assay System, and bicinchoninic acid (BCA) Protein Assay Kit were purchased from Promega (Madison, WI, USA). In addition, 30% acrylamide, 1.5 M Tris-HCl (pH 8.8), 1 M Tris-HCl (pH 6.8), 5 × SDS-PAGE protein loading buffer and 3-(4, 5-dimethyl-2-thiazolyl)-2, 5-diphenyl-2-H-tetrazolium bromide (MTT) were purchased from Beyotime (Beijing, China). Lipofectamine 2000 reagent was purchased from Invitrogen (Carlsbad, CA, USA). Dulbecco’s modified Eagle medium (DMEM) and trypsin were purchased from Hyclone (Logan, UT, USA). Roswell Park Memorial Institute (RPMI)-1640 medium was obtained from Gibco (Carlsbad, CA, USA). Fetal bovine serum (FBS) was purchased from BI (Biological Industries, Beit HaEmek, Israel).

siRNA-NC (negative control, Product number: siN0000001-1-5), siRNA-Luc (Product number: siG180802110755, Target sequence: CCGCTGAATTGGAATCCAT), siRNA-EGFP (Product number: siP04112313312) were purchased from RIBOBIO (Guangzhou, China)

SPC-A1 cells (Human lung adenocarcinoma cells, Product number: GCPC-0142094), HepG2 cells (Human liver cancer cells, Product number: SCSP-510), SMMC-7721 cells (Human liver cancer cells, Product number: GCPC-0198550), Luc-SPC-A1 cells (Luciferase human lung adenocarcinoma cells, Obtained by lentivirus infection, Lentivirus: GV260 (Ubi-MCS-firefly-Luciferase-IRES-Puromycin, Product number: LVCON101)) and EGFP-SPC-A1 cells (Enhanced green fluorescent protein human lung adenocarcinoma cells, Obtained by lentivirus infection, Lentivirus: GV492 (Ubi-MCS-3FLAG-CBh-gcGFP-IRES-puromycin, Product number: LVCON335)) were purchased from Jikai Gene Technology Co., Ltd (Shanghai, China).

*Synthesis and characterization of PEI1800-SPION*

SPION was synthesized by a co-precipitation method, as reported by Yoo (10). Successful synthesis was verified by X-ray diffractometry (XRD) (Panalytical, Netherlands). PEI1800-SPION was synthesized via a thiourea reaction between primary amine groups of PEI1800 and the isocyanate group of 3-(triethoxysilyl) propyl isocyanate (11). Successful synthesis was confirmed by Fourier transformed infrared (FTIR) spectroscopy in the range of 400~3600 cm^-1 ^(Shimadzu, Japan). The coating rate of PEI1800 on SPION was determined by thermogravimetric analysis (TGA) under dry nitrogen flow with a heating rate of 10°C/min from room temperature to 600 °C (Innuo, China). Magnetic measurements of SPION, PEI1800-SPION, and LP-PEI1800-SPION were assessed using a vibrating sample magnetometer (VSM) (Lake Shore, USA) under a circulating magnetic field in the range of -20000 to 20000 Oe.

The iron concentration in SPION and PEI1800-SPION was measured by inductively coupled plasma mass spectrometry (ICP-MS) (Thermo Fisher, USA).

*Preparation and characterization of LP-PEI1800-SPION/siRNA*

DOTAP, DOPE, and cholesterol were evenly mixed at a molar ratio of 2:1:1. Then, the mixture was dropped into HEPES buffer containing PEI1800-SPION and stirred at room temperature for 30 min. Afterward, the solution was transferred to a dialysis tube (MW 6000~8000, spectrum, USA), dialyzed in HEPES buffer for 2 h, and stored at 4 °C for further use.

siRNA was diluted in sterilized DEPC water and mixed with LP-PEI1800-SPION solution at different weight ratios. The mixture was allowed to stand at room temperature for 30 min to obtain stable LP-PEI1800-SPION/siRNA nanoparticles.

The particle sizes and Zeta potential of LP-PEI1800-SPION/siRNA nanoparticles were determined by a NanoBrook (Brookhaven, USA) at 25 °C and 90° scattering angle. The morphology was observed by transmission electron microscopy (TEM) (Shimadzu, Japan).

*Gel retardation*

The LP-PEI1800-SPION/siRNA-NC nanoparticles were prepared freshly (0.2 µg siRNA-NC) at various weight ratios (LP-PEI1800-SPION: siRNA-NC ratios of 5, 10, 20, and 40). Then, LP-PEI1800-SPION/siRNA-NC nanoparticles were thoroughly mixed with a 6 × loading buffer mix and loaded onto a 3% agarose gel with 0.5 µg/mL EtBr. After running for 60 min at 80 mV, the gel was photographed under ultraviolet light (Kezhe, China) to observe the siRNA.

*Cytotoxicity assay*

The cytotoxicity of LP-PEI1800-SPION on SPC-A1 cells, Luc-SPC-A1 cells, HepG2 cells, and SMMC-7721 cells was evaluated by MTT assay.

The cells were seeded in 96-well plates at densities of 1 × 10^4^ (SPC-A1 cells and Luc-SPC-A1 cells), 1.2 × 10^4^ (SMMC-7721 cells), and 2 × 10^4^ (HepG2 cells) and cultured at 37 °C and 95% humidity. When the cells had grown to the logarithmic stage, a serum-free medium containing different concentrations of LP-PEI1800-SPION was added to each well (0.2 μg siRNA-NC). After 24 h, MTT solution (20 μL) was added to each well, and the experiment was carried out following the manufacturer’s instructions. The absorbance (OD) was measured at 490 nm with a microplate reader (Thermo Fisher, USA). Each sample was repeated five times, and the average value was taken. The cell survival rate was calculated as follows:

Cell survival rate (%) = (OD experiment/OD control) × 100%.

*Gene silencing*

The Luc-SPC-A1 cell line is a Luciferase stable expression lung cancer cell line. To determine the optimal transfection conditions for LP-PEI1800-SPION, the silencing effects of LP-PEI1800-SPION/siRNA-Luc complexes with different ratios were investigated in Luc-SPC-A1 cells. Luc-SPC-A1 cells were seeded in 6-well plates at a density of 3.5 × 10^5^ cells/well. Then, different weight ratios of LP-PEI1800-SPION to siRNA-Luc nanoparticles in serum-free medium were added to each well. LP was used as a positive control, and naked siRNA-Luc, naked siRNA-NC, and blank medium were used as negative controls. After 6 h of culture, the nanoparticles were removed, and a fresh complete culture medium was added for another 24 h. Cells were lysed, and the protein concentration in the lysates was determined by the BCA protein assay kit. The gene silencing effects were determined using a Luciferase Reporter 1000 Assay System (Thermo Fisher, USA). The fluorescence intensity (RLU) was normalized to protein concentration (RLU/mg).

Green fluorescent protein (GFP) can emit green light under excitation, which can be observed under a fluorescence microscope (Olympus, Japan) and detected by flow cytometry (Thermo Fisher, USA), EGFP is a mutant of GFP, and its fluorescence intensity is 6 times that of GFP. In this study, SPC-A1 cells with stable EGFP expression were selected to verify the gene silencing effect of LP-PEI1800-SPION/siRNA-EGFP nanoparticles. Briefly, LP-PEI1800-SPION/siRNA-EGFP nanoparticles (with a weight ratio of LP-PEI1800-SPION to siRNA-EGFP of 20) were added to the cells in the logarithmic growth stage and cultured for 6 h. After that, the medium was replaced with a fresh complete culture medium, and cells were again placed in the incubator. The EGFP silencing effects were observed under a fluorescence microscope after being cultured for 6, 18, or 42 h and detected by flow cytometry after being cultured for 42 h.

*Prussian blue staining*

Prussian blue (potassium ferrocyanide) can react with ferric ions to form an insoluble blue compound, ferrocyanide Prussian blue. Therefore, in this study, the Prussian Iron Stain Kit was used to investigate the cellular uptake of LP-PEI1800-SPION. Briefly, cells in the logarithmic growth phase were seeded in a 6-well plate at a density of 5 × 10^5^ cells/well (HepG2 cells) or 3.5 × 10^5^ cells/well (SMMC-7721 cells), treated with LP-PEI1800-SPION/siRNA-Luc nanoparticles (with a weight ratio of LP-PEI1800-SPION to siRNA-Luc of 20), and cultured for 6 h. Hereafter, the cells were stained using the Prussian Blue Iron Stain Kit and observed under the microscope.

*In-vitro MRI*

To study whether LP-PEI1800-SPION can be used as an MRI contrast agent, the intensity of the magnetic resonance signal was evaluated after the cellular uptake of the contrast agent. Cells in the logarithmic growth phase were seeded in a 6-well plate at a density of 5 × 10^5^ cells/well (HepG2 cells) or 3.5 × 10^5^ cells/well (SMMC-7721 cells) and cultured for 18–22 h. Then the cells were treated with 1, 2, or 4 μg siRNA-Luc nanoparticles (with a weight ratio of LP-PEI1800-SPION to siRNA-Luc of 20) for 6 h. Cells were collected, resuspended in 0.3 mL 0.9% agarose solution, and detected by a 3.0 T MRI scanner (Siemens, Germany). Setting parameters were as follows: TR (repetition time), 3700 ms; TE (echo time), 117 ms; FOV (field of view), 12 × 12 cm; layer thickness, 4 mm.

*Statistical analysis*

All the data in this text were statistically analyzed using GraphPad Prism 5.0, and *P <* 0.05 indicated significant differences between the groups.

## Results

**Figure 1:**
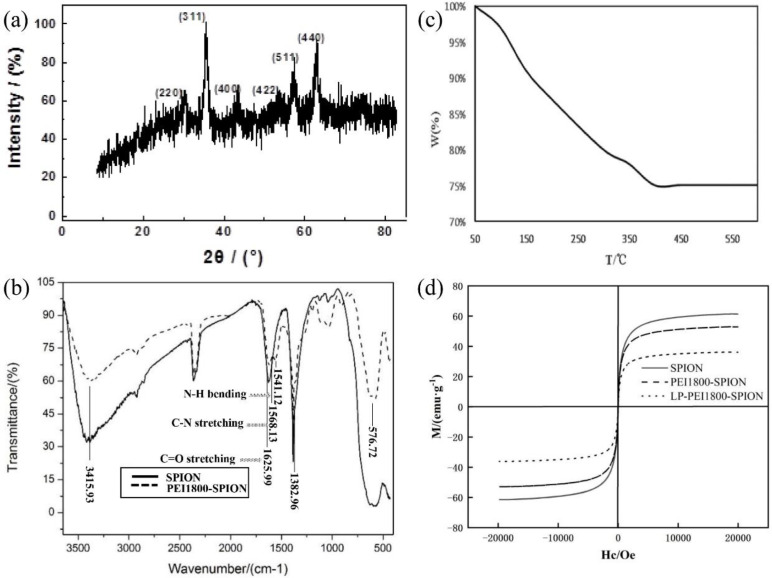
Characterization of SPION, PEI1800-SPION and LP- PEI1800-SPION, (a) SPION X diffraction pattern (b) FTIR spectrum of SPION and PEI1800-SPION (c) Thermogravimetric analysis of PEI1800-SPION (d) Magnetic hysteresis loops of SPION, PEI1800-SPION and LP- PEI1800-SPION

The SPION crystal type was analyzed by XRD. The six characteristic peaks of the X-ray diffraction pattern of SPION (2 theta = 30.4°, 35.7°, 43.4°, 53.4°, 57.4°, and 62.7°) corresponded to the cubic phase of the Fe_3_O_4_ (220), (311), (400), (422), (511), and (440) crystal plane, respectively, after comparison with the standard card 01-088-0866, showing that the product obtained was Fe_3_O_4 _(Figure 1a). The structure of PEI1800-SPION was verified by FTIR spectroscopy (Figure 1b). The characteristic absorption peaks of SPION are at 576.72, 1382.96, and 3415.93 cm^−1^. At 1541.12 and 1568.13 cm^−1^, we observed the absorption peaks obtained by the N-H bending vibration and the C-N stretching vibration, respectively. The stretching vibration peak of C=O in the amide bond was at 1625.99 cm^−1^, showing that PEI1800-SPION was successfully synthesized.

The coating rate of PEI on SPION was determined by TGA. In order to prevent the oxidation of Fe_3_O_4_ in the air, TGA was conducted under nitrogen protection conditions. The weight loss curve of PEI1800-SPION is shown in Figure 1c. The weight of PEI1800-SPION decreases gradually when the temperature rises from 50°C to 400°C. When the temperature rises above 400 °C, the weight is basically unchanged. Therefore, the weight encapsulation rate of PEI on the surface of SPION is about 24.9%. Moreover, no weight gain was observed according to the curve, indicating that after PEI1800-modified SPION, the surface of PEI1800-SPION was not easily oxidized during the detection process, and its stability was improved. Figure 1d displays the superparamagnetic property of SPION, PEI1800-SPION, and LP-PEI1800-SPION in the powder state at room temperature. The saturation magnetization (*δs*) was determined to be 61.42 emu·g^-1^, 52.88 emu·g^-1 ^and 36.24 emu·g^-1 ^for SPION, PEI1800-SPION, and LP-PEI1800-SPION, respectively. According to the reduction in *δs* value of PEI1800-SPION and LP-PEI1800-SPION compared to SPION. Differences may arise from the proportion of iron oxide phases and the modification of the polymer and LP encapsulation (12).

**Figure 2:**
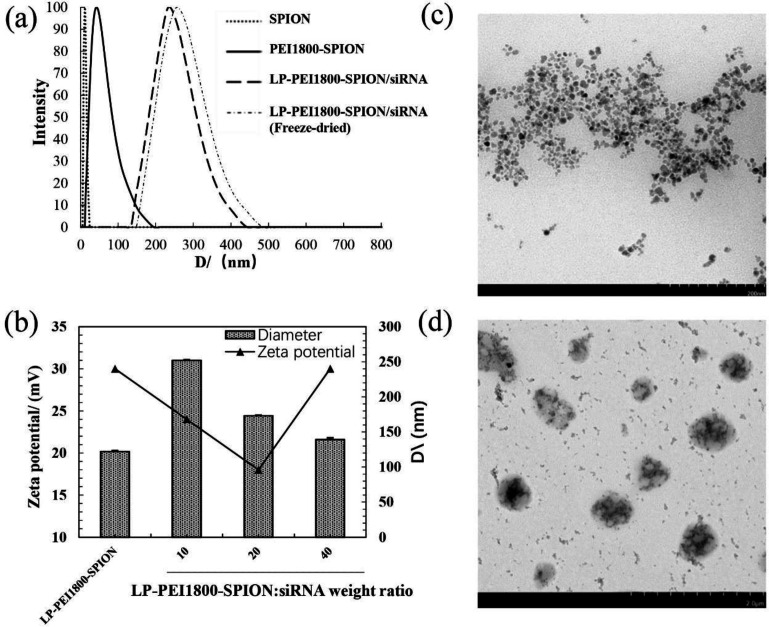
Characterization of (a) Particle sizes of SPION, PEI1800-SPION and LP-PEI1800-SPION (b) Particle sizes and Zeta potential values of LP-PEI1800-SPION/siRNA at various weight ratios. Transmission electron microscope image of (c) PEI1800-SPION and (d) LP-PEI1800-SPION/siRNA

Particle sizes and Zeta potential are two important characteristics of nanoparticles. They affect many *in-vivo* processes, such as transfection efficiency, pharmacokinetics, and distribution in the body. The particle sizes of the LP-PEI1800-SPION/siRNA nanoparticles are between 130 and 250 nm. As shown in Figure 2b, after combining LP-PEI1800-SPION with siRNA at different weight ratios, the particle sizes of the nanoparticles gradually decreased as the weight ratio increased. Probably due to the increase in weight ratio, the combination of cationic polymer and siRNA becomes tighter. The Zeta potential has a tendency to first decrease and then increase. When the weight ratio is 20, the Zeta potential of the composite surface is at least 18.06 ± 1.72 mV, and the particle size is 174.33 ± 1.15 nm.

It can be seen from the TEM image (Figure 2d) that the LP-PEI1800-SPION/siRNA nanoparticles are spherical and ellipsoidal, and their size is about 250 nm, basically consistent with the particle size measured by the particle size analyzer.

*Gel retardation*

**Figure 3:**
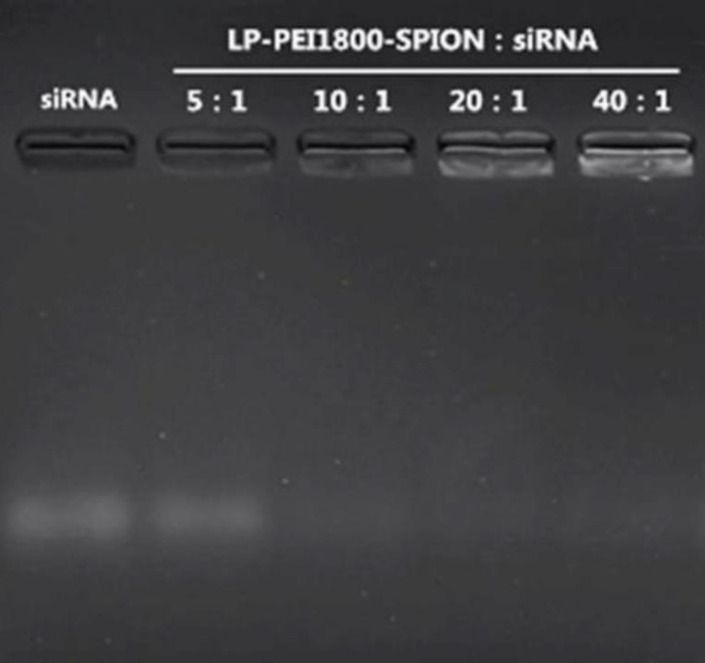
Agarose gel electrophoresis images of LP-PEI1800-SPION/siRNA at various weight ratios from 5 to 40, and naked siRNA was used as a control

A suitable gene carrier can effectively compress siRNA and protect it from nuclease degradation. Agarose gel electrophoresis can be used to investigate the combination of carrier and siRNA. As shown in Figure 3, as the weight ratio of LP-PEI1800-SPION to siRNA increases, the electrophoretic bands of siRNA are gradually reduced. The siRNA band is no longer displayed in the gel when the weight ratio is 10, indicating that the siRNA has been stably bound to the carrier and blocked in the sample hole.

*Cytotoxicity experiment*

**Figure 4:**
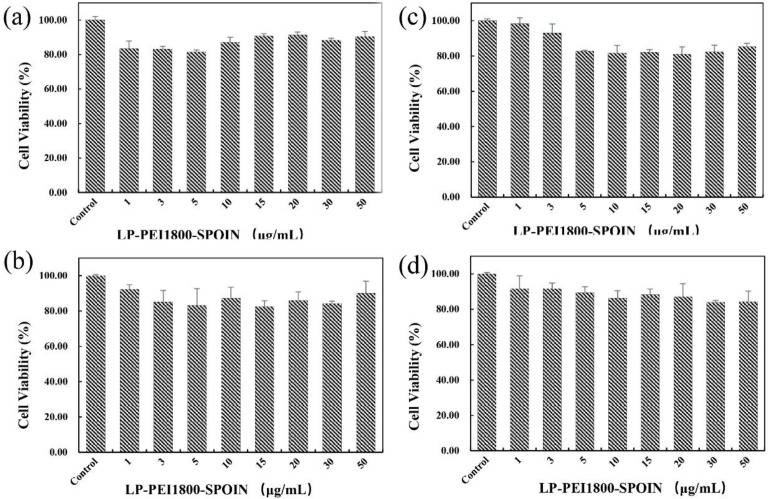
Cell viability measurement with MTT assay after LP-PEI1800-SPION treatment ranging from 1 to 50 μg/mL (the cells with no treatment was used as a control) in four different cell lines for 24 h (a) SPC-A1 (b) HepG2 (c) Luc-SPC-A1 and (d) SMMC-7721 (mean ± SD, n = 5)

The cytotoxic effects of LP-PEI1800-SPION on SPC-A1 cells, Luc-SPC-A1 cells, HepG2 cells, and SMMC-7721 cells were determined by MTT assay. As shown in Figure 4, the cell survival rate is above 80% in the range of 1–50 μg/mL of LP-PEI1800-SPION, indicating that the gene carrier LP-PEI1800-SPION is little toxic to cells at high concentrations.

*Gene silencing*

**Figure 5:**
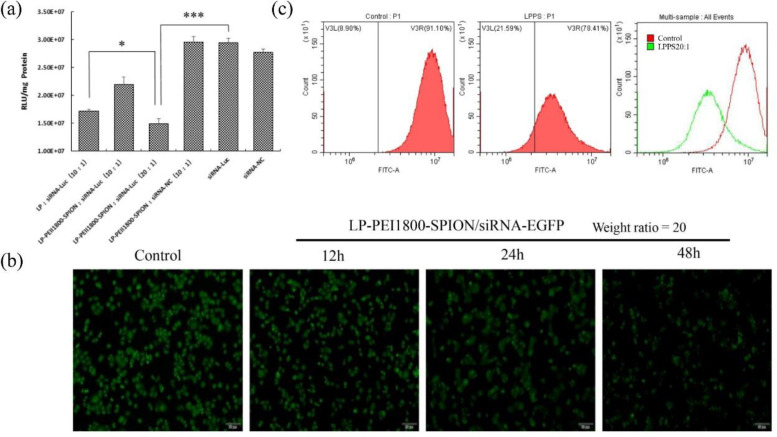
Gene silencing effect of LP-PEI1800-SPION/siRNA, (a) Transfection efficiency of LP-PEI1800-SPION/siRNA-Luc on Luc-SPC-A1 cells ^(^^***^P＜0.001 compared with naked siRNA-Luc group, ^*^P＜0.05 compared with LP/siRNA-Luc group). (b) Green fluorescent protein silencing effect on EGFP-SPC-A1 cells by LP-PEI1800-SPION/siRNA-EGFP at various times under a fluorescence microscope (the weight ratio of LP-PEI1800-SPION to siRNA-EGFP was 20. The cells with no treatment was used as a control) (c) Flow cytometry analysis of green fluorescent protein silencing efficiency in EGFP-SPC-A1 cells treated with LP-PEI1800-SPION/siRNA-EGFP for 48 h (the weight ratio of LP-PEI1800-SPION to siRNA-EGFP was 20)

In order to achieve the best transfection efficiency of the nanoparticles, the weight ratio of the carrier and siRNA was screened. As shown in Figure 5a, when the weight ratio of LP-PEI1800-SPION/siRNA-Luc is 20, the nanoparticle significantly reduces the expression of the Luciferase reporter gene. Its silencing efficiency reaches 47% (*P *< 0.001), which is significantly higher than that of the LP/siRNA-Luc group (*P *< 0.05). This indicated that the addition of PEI1800-SPION improved the transfection efficiency of gene vectors. The LP-PEI1800-SPION/siRNA-NC group and naked siRNA-NC group exhibited no significant silencing effects.

To verify the transfection efficiency of LP-PEI1800-SPION, the silencing effect of LP-PEI1800-SPION/siRNA-EGFP nanoparticles on EGFP-SPC-A1 cells was examined by fluorescence microscopy. The results are shown in Figure 5b. The untreated EGFP-SPC-A1 cells emitted obvious green fluorescence, which was weakened after transfection with PEI1800-SPION/siRNA-EGFP for 24 h. After 48 h, the silencing effect reached its maximum, indicating siRNA-EGFP can interfere with the mRNA synthesis of target genes and affect the expression of EGFP protein, proving that LP-PEI1800-SPION can safely and effectively transport siRNA into cells and enhance the silencing effect.

We also checked the transfection efficacy by flow cytometry. EGFP is a GFP mutant with a maximum excitation wavelength of 488 nm and a maximum emission wavelength of 508 nm. We selected the FITC channel to analyze the expression of GFP. The results are shown in Figure 5c. After EGFP-SPC-A1 cells are treated with LP-PEI1800-SPION/siRNA-EGFP nanoparticles, the fluorescence intensity curve shifts significantly to the left, and the fluorescence intensity of the FITC channel drops from 91.10% to 78.41%. This was consistent with the results observed by fluorescence microscopy, which further verified that LP-PEI1800-SPION successfully delivered siRNA into the cells and interfered with the expression of GFP.

*Prussian blue staining*

**Figure 6:**
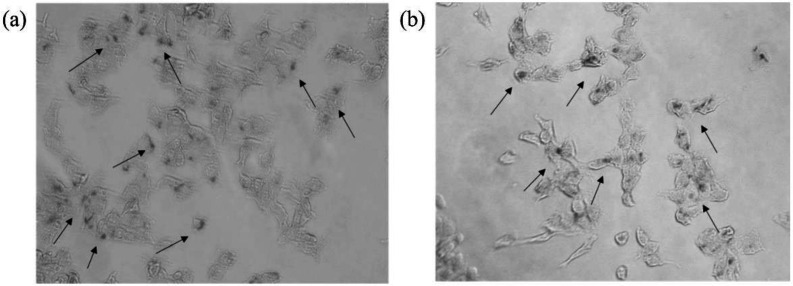
Prussian blue staining images of cells incubation with LP-PEI1800-SPION/siRNA-Luc at the weight ratio of 20 for 6 h (a) HepG2 and (b) SMMC-7721 (The arrow in the figure points to the SPION nanoparticles taken up in the cells)

The cellular uptake of LP-PEI1800-SPION was investigated by the Prussian Iron Stain Kit. The results are shown in the Figure 6. After LP-PEI1800-SPION/siRNA was administered and cells were incubated for 6 h, multiple blue-stained iron particles were seen in the cells, proving that the cells successfully took up the LP-PEI1800-SPION/siRNA nanoparticles. Studies have shown that the cellular uptake efficiency of SPION is closely related to the biological characteristics of the cell line and the surface coating of nanoparticles. As shown in the Figure 6, there are slightly more iron particles in HepG2 cells than in SMMC-7721 cells, which may be due to the strong uptake ability of HepG2 cells.

*In-vitro MRI*

**Figure 7:**
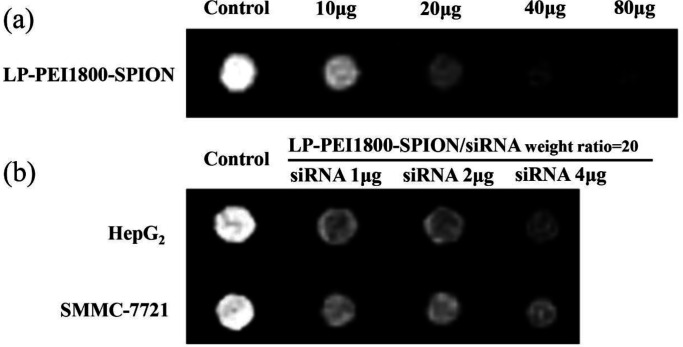
Images T2-weighted MRI with (a) LP-PEI1800-SPION at various weight ranging from 10 to 80 μg in PBS and (b) LP-PEI1800-SPION/siRNA-Luc at various weight ratios from 1 to 4 μg of siRNA-Luc in HepG2 and SMMC-7721 cells (TE = 117 ms. The cells with no treatment was used as a control)

SPION is an MRI T2 negative contrast agent, which can reduce T2 signal intensity and make the image appear darker. The MRI results of HepG2 and SMMC-7721 cells incubated *In-vitro* with LP-PEI1800-SPION/siRNA are shown in the Figure 7. Compared with the blank control group, with increasing LP-PEI1800-SPION/siRNA concentration, the MRI images became darker, with significant differences compared with the blank control group, showing that the LP-PEI1800-SPION gene vector has the potential to serve as a contrast agent. This requires further exploration in clinical practice.

## Discussion

Gene silencing is classified according to its mechanism of action as transcriptional gene silencing (TGS) and post-transcriptional gene silencing (PTGS). PTGS, which is mediated by siRNA at the RNA level, is more common than TGS, which takes place at the DNA level, and is one of the current research hotspots. Transfection with a specific siRNA sequence can achieve the degradation of mRNA, thereby inhibiting protein expression. siRNA drugs can complete the silencing effect in the cytoplasm, are not integrated into the target cell DNA, and do not permanently change the genome, allowing treatment strategies to be easy to control and relatively safe (13). Like all gene drugs, the biggest problem of siRNA is its easy degradation, weak transmembrane ability, and short half-life *in-vivo*. It is particularly important to choose a suitable carrier to deliver it to the target cell safely and efficiently to complete the silencing effect. Studies have shown that cationic liposomes have a high transfection efficiency, but this is still significantly lower than that of viral vectors, and cationic liposomes are prone to rapid inactivation in the presence of serum due to their instability (14, 15).

PEI has a good pH buffering capacity and can effectively promote the release of DNA/siRNA into the cytoplasm, making it one of the classic and most effective non-viral polymeric gene carriers. However, as the molecular weight of PEI increases, its transfection efficiency increases, but its toxicity also increases. The difficulty of balancing between transfection efficiency and toxicity limits the application of PEI.

Studies have shown that the transfection efficiency of low-molecular-weight PEI (MW: 0.6–1.8 kDa) is very low. However, when it is combined with (cationic or anionic) liposomes, they can exert a synergistic effect in introducing siRNA into cells. Zhang (16) used GFP-siRNA-load BERA (bioengineered RNAi agents) as a model drug to evaluate the silencing efficiency of the liposome-PEI nanocomposite. The results showed that the nanocomposite could protect siRNA from nuclease degradation and downregulate the expression of GFP mRNA in a mouse orthotopic hepatocellular carcinoma model (60%), which was better than the silencing efficiency of the IVJ-PEI (*in-vivo*-jet PEI) transfection reagent (36%). Lee (17) combined PEI with cationic liposomes and transfected cells with P53 (tumor suppressor gene) antisense oligonucleotide, which could effectively inhibit the synthesis of P53 protein in HepG2 cells (68%) and 2215 cells (43%). The transfection efficiency of PEI in combination with liposomes was more than 10 times higher than that of polymer or liposome alone.

In the present study, similar results were obtained. Cationic liposome LP with DOTAP, DOPE, and cholesterol at a molar ratio of 50:25:25 was constructed by an ethanol injection method and combined with PEI1800-SPION to form LP-PEI1800-SPION nanoparticles for siRNA delivery. SPION has the characteristics of a large constant magnetic moment and rapid response to the magnetic field. Due to the high energy on the surface, SPION is unstable and easy to oxidize in the air, which will cause the accumulation of SPION and the loss of partial magnetism (18, 19). The researchers provided a solution to these problems by using polymer coatings to modify SPION (18). As in this article, combining PEI and SPION through thiourea reaction can not only improve the stability of SPION but also give it more functions, such as gene delivery (20, 21). From the gene transfection experiment (Figure 5a), we can observe when the weight ratio of LP-PEI1800-SPION and siRNA was 20, the gene silencing efficiency of the Luciferase reporter gene in the cells reached 47%, which was higher than that of the single LP group. This might be due to the co-transfection effect between PEI and the liposome. The phospholipid bilayer structure in the complex is similar to the cell membrane, which makes the complex enter the cell easily (22). In addition, due to the DOPE component in the prescription, it helps to destroy the endosome and transport nucleic acids to the cytoplasm, which is beneficial for gene transfection (23). The liposome promotes the uptake of nanoparticles by cells, while PEI accelerates the escape of siRNA from the endosome through its proton sponge effect.

The particle size and Zeta potential of the carrier and DNA complex are around 170 nm and 18 mV. Studies have shown that positively charged delivery systems will lead to greater cell internalization than other charged systems (24). The toxicity of gene carriers has always been discussed; the hydrophilic group structure of cationic lipids, the molecular weight of cationic polymers PEI, and the degree of branching are the main factors affecting the toxicity of the carrier (22). Some studies have provided relevant strategies to reduce gene delivery vectors toxicity by introducing chemical groups on the structure of cationic lipids or linking low molecular weight PEI with beta-cyclodextrin, polyethylene glycol (PEG), acid-labile imine, etc. (25, 26). In this study, the lower molecular weight PEI1800 was selected to prepare LP-PEI1800-SPION, which shows a low toxicity performance in the range of 1-50 μg/mL; the *in-vivo *toxicity of this carrier will be further studied in the following animal experiments.

With the rapid development of medical technology, people expect to monitor the treatment process of diseases in real-time. Wrapping SPION in lipid polycationic nanoparticles allows it to be targeted to tumor sites and displayed on MRI so as to monitor the treatment process of the disease in real-time and adjust the treatment plan timely, such as selecting the optimal time interval of drug administration, monitoring the tumor size, and judging the progress of drug treatment. The *in-vitro* MRI results in this study showed that LP-PEI1800-SPION could be effectively internalized by cells and significantly reduce the T2 signal intensity, indicating that it could also be used as an MRI contrast agent to improve the sensitivity of MRI detection. In conclusion, this dual-functional nanoparticle can combine MRI with gene therapy to achieve *in-vivo* visualization of gene delivery, release, and accumulation. The disease process can be studied more accurately through tissue imaging so as to realize individualized treatment and precision medicine.

SPION in LP-PEI1800-SPION also has a variety of applications in tumor treatment, its magnetic targeting property can be used to deliver drugs to tumor cells (27), and its thermal conversion property can also be used to kill tumor cells (28), the effect of LP-PEI1800-SPION in these aspects need to be further explored and verified.

## Conclusion

In this experiment, an MRI visualization lipid polycationic siRNA delivery vector (LP-PEI1800-SPION) was prepared, which enables siRNA drugs to achieve high-efficiency protein silencing and accuracy at the same time diagnosis and monitoring the disease treatment progress through MRI imaging to achieve the best therapeutic effect. This will provide a preliminary experimental basis and theoretical basis for the study of visualized nano gene carriers.

## Author contributions

JS and YZ conducted experiments and wrote the manuscript. GJ reviewed this manuscript. YJ searched the literature. JQ designed the study. All authors have read and approved the ﬁnal manuscript.

## Disclosure

All authors declare no competing financial interests.

## References

[1] Mattiuzzi C, Lippi G (2019). Current Cancer Epidemiology. J. Epidemiol. Glob. Health.

[2] Urruticoechea A, Alemany R, Balart J, Villanueva A, Vinals F, Capella G (2010). Recent advances in cancer therapy: an overview. Curr. Pharm. Des..

[3] Chen X, Chen L, Li D (2019). Research progress of gene therapy in clinical application. Sheng Wu Gong Cheng Xue Bao.

[4] Subhan MA, Torchilin VP (2019). Efficient nanocarriers of siRNA therapeutics for cancer treatment. Transl Res..

[5] Li Y, Huang X, Lee RJ, Qi Y, Wang K, Hao F, Zhang Y, Lu J, Meng Q, Li S, Xie J, Teng L (2016). Synthesis of Polymer-Lipid Nanoparticles by Microfluidic Focusing for siRNA Delivery. Molecules.

[6] Hadinoto K, Sundaresan A, Cheow WS (2013). Lipid-polymer hybrid nanoparticles as a new generation therapeutic delivery platform: a review. Eur. J. Pharm. Biopharm..

[7] Pinnapireddy SR, Duse L, Strehlow B, Schafer J, Bakowsky U (2017). Composite liposome-PEI/nucleic acid lipopolyplexes for safe and efficient gene delivery and gene knockdown. Colloids Surf. B Biointerfaces.

[8] Wu Z, Xu XL, Zhang JZ, Mao XH, Xie MW, Cheng ZL, Lu LJ, Duan XH, Zhang L (2017). M and Shen J. Magnetic cationic amylose nanoparticles used to deliver survivin-small interfering rna for gene therapy of hepatocellular carcinoma in-vitro. Nanomaterials.

[9] Shen M, Gong F, Pang P, Zhu K, Meng X, Wu C, Wang J, Shan H, Shuai X (2012). An MRI-visible non-viral vector for targeted Bcl-2 siRNA delivery to neuroblastoma. Int. J. Nanomedicine.

[10] Yoo MK, Park IK, Lim HT, Lee SJ, Jiang HL, Kim YK, Choi YJ, Cho MH, Cho CS (2012). Folate-PEG-superparamagnetic iron oxide nanoparticles for lung cancer imaging. Acta Biomater..

[11] Kim, YK, Zhang M, Lu JJ, Xu F, Chen BA, Xing L, Jiang HL (2016). PK11195-chitosan-graft-polyethylenimine-modified SPION as a mitochondria-targeting gene carrier. J. Drug Target..

[12] Perecin CJ, Tirich BM, Nagamine LC, Porto G, Rocha FV, Cerize, NN, Varanda LC (2021). Aqueous synthesis of magnetite nanoparticles for magnetic hyperthermia: formation mechanism approach. high water-dispersity and stability. Colloids Surf. A..

[13] Resnier P, Montier T, Mathieu V, Benoit JP, Passirani C (2013). A review of the current status of siRNA nanomedicines in the treatment of cancer. Biomaterials.

[14] Dokka S, Toledo D, Shi X, Castranova V, Rojanasakul Y (2000). Oxygen radical-mediated pulmonary toxicity induced by some cationic liposomes. Pharm. Res..

[15] Lee H, Williams SK, Allison SD, Anchordoquy TJ (2001). Analysis of self-assembled cationic lipid-DNA gene carrier complexes using flow field-flow fractionation and light scattering. Anal. Chem..

[16] Zhang QY, Ho PY, Tu MJ, Jilek JL, Chen QX, Zeng S, Yu AM (2018). Lipidation of polyethylenimine-based polyplex increases serum stability of bioengineered RNAi agents and offers more consistent tumoral gene knockdown in-vivo. Int. J. Pharm..

[17] Lee CH, Ni YH, Chen CC, Chou C, Chang FH (2003). Synergistic effect of polyethylenimine and cationic liposomes in nucleic acid delivery to human cancer cells. Biochim. Biophys. Acta.

[18] Ditsch A, Laibinis PE, Wang DI, Hatton TA (2005). Controlled clustering and enhanced stability of polymer-coated magnetic nanoparticles. Langmuir.

[19] Lu AH, Salabas EE, Schüth F (2007). Magnetic nanoparticles: synthesis, protection, functionalization, and application. Angew. Chem. Int. Ed..

[20] Yang Z, Duan J, Wang J, Liu Q, Shang R, Yang X, Dou K (2018). Superparamagnetic iron oxide nanoparticles modified with polyethylenimine and galactose for siRNA targeted delivery in hepatocellular carcinoma therapy. Int. J. Nanomed..

[21] Mulens AV, Rojas JM, Sanz OL, Portilla Y, Pérez YS, Barber DF (2019). Polyethylenimine-coated superparamagnetic iron oxide nanoparticles impair in-vitro and in-vivo angiogenesis. Nanomedicine.

[22] Lv H, Zhang S, Wang B, Cui S, Yan J (2006). Toxicity of cationic lipids and cationic polymers in gene delivery. J. Control. Release.

[23] Du Z, Munye MM, Tagalakis AD, Manunta MD, Hart SL (2014). The role of the helper lipid on the DNA transfection efficiency of lipopolyplex formulations. Sci. Rep..

[24] Hajj KA, Whitehead KA (2017). Tools for translation: non-viral materials for therapeutic mRNA delivery. Nat. Rev. Mater..

[25] Meekel AA, Wagenaar A, S misterová J, Kroeze JE, Haadsma P, Bosgraaf B, Engberts JBFN (2000). Synthesis of pyridinium amphiphiles used for transfection and some characteristics of amphiphile/DNA complex formation. Eur. J. Inorg. Chem..

[26] Tang GP, Guo HY, Alexis F, Wang X, Zeng S, Lim TM, Wang S (2006). Low molecular weight polyethylenimines linked by β‐cyclodextrin for gene transfer into the nervous system. J. Gene Med..

[27] Zhuang M, Du D, Pu L, Song H, Deng M, Long Q, Rao L (2019). SPION-decorated exosome delivered BAY55‐9837 targeting the pancreas through magnetism to improve the blood GLC response. Small..

[28] Patil SY, Torino E, De SF, Ponsiglione AM, Chhabria V, Ahmed W, Mercer T (2020). Biocompatible superparamagnetic core-shell nanoparticles for potential use in hyperthermia-enabled drug release and as an enhanced contrast agent. Nanotechnology.

